# Long-Range Redox and Water Activation at Metal–Water Interfaces with Ferroelectric Ordering

**DOI:** 10.1021/acs.jctc.5c00814

**Published:** 2025-07-24

**Authors:** Arthur Hagopian, Jean-Sébastien Filhol, Tobias Binninger

**Affiliations:** † Leiden Institute of Chemistry, 4496 Leiden University, Leiden 2333CC, The Netherlands; ‡ ICGM, CNRS, 173567 University of Montpellier, Montpellier 34293, France; § Theory and Computation of Energy Materials (IET-3), 233859 Institute of Energy Technologies, Forschungszentrum Jülich GmbH, 52425 Jülich, Germany

## Abstract

The molecular structure of water has profound influence on electron transfer and redox processes at metal–water interfaces. While ab initio molecular dynamics simulations provide an accurate description of the interfacial structure, the respective computational cost is often prohibitive. Static simulations using a few ordered water layers can serve as a pragmatic alternative maintaining an explicit description of molecular interactions at an affordable computational cost. We here study the coupling between electronic and structural degrees of freedom at ferroelectrically ordered metal–water interfaces. With increasing number of ice-like water layers, we observe a long-range transfer of electrons between the metal’s Fermi level and HOMO/LUMO states of the outermost water molecules, mediated by ordered solvent dipole layers. Our findings reveal limitations of the applicability of the ordered interface model and reveal a strong coupling between ferroelectric ordering and long-range (auto)­redox phenomena at dipolar solvent structures, shedding new light onto the long-standing question on the existence and stability of ferroelectric ice. Implications for the activation of water molecules in electrocatalytic reactions at charged metal–water interfaces are suggested.

## Graphical abstract

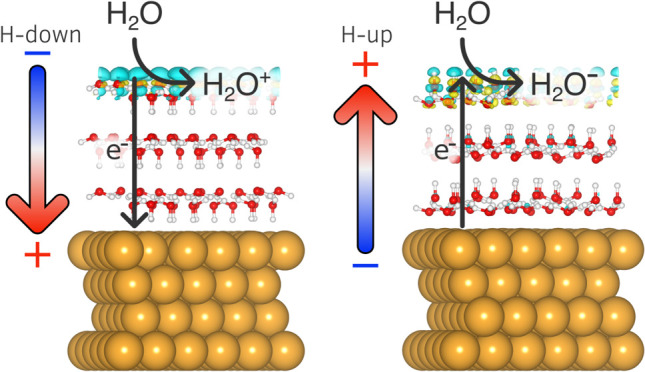

## Introduction

Metal–water interfaces play a key role in various fields of research, ranging from corrosion to electrocatalysis. Their electrochemical behavior is strongly influenced by the local structure of the solvent. Specific interactions between water molecules and the metal surface can result in structural ordering of the interfacial water layer. The hexagonal structure and interatomic distances of the (111) surfaces of platinum and gold, for instance, closely match the structure of water bilayers in the common hexagonal ice I_h_ configuration.^1^ The ice-like bilayer model is thus frequently used to describe adlayers of water on metal surfaces, although experimental results often indicate more complicated adsorption structures.^2^

Of particular relevance is the question of a preferential orientation of water dipoles at the interface, which can have drastic consequences for the work function, or, in electrochemical terms, the potential of zero charge (PZC), of the metal–water interface.^3,4^ The H-up and H-down configurations represent two limiting cases, where out-of-plane hydrogen atoms of the water bilayer either point outward or toward the metal surface, respectively. Due to the opposite alignment of the molecular dipoles, the H-up and H-down configurations can lead to a difference in work function of few electronvolts, as obtained from density functional theory (DFT) calculations.^1,3–5^ However, ab initio molecular dynamics (AIMD) simulations of water films on metal surfaces at room temperature have shown no, or only minor, preferred orientation of water dipoles at the uncharged interface,^3,6,7^ although the ice-like hexagonal structure of the first water layer remained preserved on certain metal surfaces, such as Pt(111), with stronger metal–water interactions.^3^ Likewise, experimental results for the work function of metal surfaces covered with (sub)­monolayers of water under ultra-high vacuum (UHV) conditions at low temperatures showed values well in between the H-up and H-down limiting cases, indicating net zero dipole moment of the water adlayer.^1,8,9^ It should be noted that, similarly, in the common ice I_h_ phase, water bilayers are net unpolarized due to statistical distribution of molecular orientations, giving rise to paraelectric behavior^10,11^ and nonzero residual entropy.^12^

However, a proton-ordered phase of ice, designated as ice XI, has been obtained at temperatures below 72 K in the presence of KOH as a dopant, which was required to facilitate the reorientation of water molecules and the transition to the proton-ordered configuration.^11,13–15^ The structure of ice XI is similar to the one of ice I_h_, however, with ordered molecular orientations, which has been interpreted as a ferroelectric phase with a nonvanishing net dipole moment. The assumed proton-ordered bilayers of ice XI directly correspond to the H-up or H-down configurations commonly considered in models of water adlayers on metal surfaces.^2^ Films of pure ice XI have been obtained on Pt(111) surfaces at temperatures between 120 and 137 K.^16^

While the existence of a ferroelectric ice XI phase has been confirmed by DFT calculations,^17^ other groups have questioned the stability of ferroelectric ordering and rather proposed an antiferroelectric arrangement of dipoles with a zero net polarization.^18–20^ Parkkinen et al. have pointed out that periodic bulk calculations provide an incorrect picture of the suggested stability of the ferroelectric phase because of the cancellation of the depolarization field by periodic Poisson solvers.^20^ They argued that the ferroelectric configuration is dramatically destabilized when taking into account the energy contribution of the depolarization field, thus favoring an effectively antiferroelectric ordering of water dipoles in ice XI. This argument is supported by experimental results showing net ferroelectric alignment of water dipoles of only about 1% and less for amorphous ice films at low temperatures.^21,22^ On the other hand, in the presence of ionic impurities, such as KOH or NaOH, the depolarization field could be compensated by ionic surface charges.^20^

Compensation charges can also be generated intrinsically. In their DFT study of ice XI films at various degrees of ferroelectric ordering, Parkkinen et al.^20^ observed spontaneous dissociation of surface water molecules into OH^–^/H_3_O^+^ pairs, which they interpreted as being caused by the large depolarization field at increased net polarization of the film. The OH^–^ and H_3_O^+^ subsequently migrated to opposite surfaces of the ice slab to screen the depolarization field and stabilize the ferroelectrically ordered state. In an experimental study of ice films grown on Pt(111) substrate, Nie et al.^23^ proposed a model where the depolarization field bends the electronic band structure of the ice film downward, with the consequence that the ice conduction band edge crosses and falls below the Pt Fermi level at a short distance from the substrate. As a result, electrons tunnel from the Pt substrate into the ice film, getting trapped at protonic defects and building up a negative space charge that compensates the depolarization field. A similar charge injection mechanism was proposed by Sugimoto et al.,^24^ however, with opposite sign. Based on sum-frequency generation spectroscopy, they concluded a net H-down polarization of the heteroepitaxial ice film on Pt(111), causing an upward bending of the electronic band structure and injection of holes from the substrate into the valence band of the ice film, corresponding to the formation of water cations, H_2_O^+^. By reaction with adjacent water molecules, the latter would subsequently transform into hydronium cations, H_2_O^+^ + H_2_O → H_3_O^+^ + ^•^OH, which would redistribute to screen the depolarization field and stabilize the ferroelectric ordering. While in certain details the models proposed by Nie et al.,^23^ Parkkinen et al.,^20^ and Sugimoto et al.^24^ differ, the following common features arise: (1) The ferroelectrically ordered configuration of ice XI is unstable due to the strong depolarization field; (2) the latter favors the generation of mobile charged species in the form of electrons, holes, or dissociated water molecules (OH^–^/H_3_O^+^) that redistribute in a way to screen the depolarization field; (3) while such screening processes effectively stabilize the ferroelectric state, the generation and redistribution of charged defects also randomizes the proton ordering in the ice crystal to a certain extent—stabilization and destruction of the ferroelectric ordering are thus intrinsically coupled.

In the present work, we confirm the electron and hole injection mechanisms proposed by Nie et al.^23^ and Sugimoto et al.^24^ by DFT calculations of gold-supported ferroelectrically ordered ice XI films. Moreover, we demonstrate that similar charge separation mechanisms are expected in free-standing ferroelectric ice slabs by long-range autoredox processes between molecular HOMO and LUMO states, resulting in the formation of water cations H_2_O^δ+^ and anions H_2_O^δ−^ at opposite surfaces of the ice crystal. While we do not attempt to evaluate the relative stability of polarized vs unpolarized ice films, or the preferred direction of polarization on metallic substrates, our study refines previously proposed models for the compensation of the depolarization field due to intrinsic charge separation and dissociation processes. This insight is not only relevant for the debate on the existence of ferroelectric ice, but also for the understanding of the coupling between water orientation and water activation at electrocatalytic interfaces. We also discuss that ferroelectric and ferromagnetic materials, while bearing some similarities in their behavior, can strongly differ in their coupling to redox activation.

## Methods

The coupling of structural and electronic properties of ferroelectric ice films, both free-standing and heteroepitaxially supported on a Au(111) substrate, was investigated by DFT calculations using the Vienna Ab initio Simulation Package (VASP).^25^ The electronic wave functions were expanded in a plane-wave basis set with a kinetic energy cutoff of 400 eV. Projector augmented wave (PAW) pseudopotentials as implemented in VASP were employed. The exchange–correlation effects were accounted for using the generalized gradient approximation (GGA) with the functional of Perdew, Burke, and Ernzerhof (PBE).^26^ van der Waals dispersion interactions were included by using the semiempirical correction proposed by Grimme.^27^ A convergence criterion of 1 × 10^–6^ and 1 × 10^–5^ eV was set for electronic self-consistent iterations and ionic relaxations, respectively. A Fermi smearing with a width of 0.05 eV was used to minimize artificial broadening of the electronic states near the Fermi level.

Free-standing proton-ordered ice slabs were constructed from the relaxed bulk cell of ice XI (dipolar-ordered form of ice I_h_) with an in-plane hexagonal lattice constant of 7.60 Å. A single water bilayer structure was obtained by cleaving the bulk cell along the [001] plane. The in-plane lattice constant and atomic positions of the bilayer were scaled to match the Au(111) substrate used in this work, i.e., compression of 3%, and duplicated in *x* and *y* in order to give a better representation of the degrees of freedom of the water molecules. A cell made of two water bilayers in vacuum was first optimized in order to refine the interbilayer distance. Note that the *z*-position of a bilayer was determined as the average of the *z*-positions of all atoms contained in the bilayer. An interbilayer distance of 4.0 Å was found and used to build the initial geometry of the ice slabs containing up to 6 water bilayers.

Furthermore, 5 × 5 hexagonal Au(111) slabs with 4 atomic layers were constructed from the relaxed fcc cell of bulk gold with a lattice parameter of 4.10 Å. Heteroepitaxial bilayers of proton-ordered ice XI were placed at a distance of 2.7 Å from the Au(111) substrate in both H-up and H-down orientations.^28^ A vacuum region of 42 Å was inserted in the *z*-direction of the cell in order to ensure that a vacuum width of at least 17 Å remained for the thickest ice slabs (6 bilayers) placed above the gold surface. The total dimensions of the simulation cells (with and without the Au substrate) were 14.7 × 14.7 × 49.2 Å^3^. The initial geometries for all free-standing and Au(111)-supported ice slabs are shown in Figures S1 and S2, respectively. Due to the large dimensions of the simulation cells, both free-standing and Au-supported ice slabs were initially relaxed using a 1 × 1 × 1 Γ-centered Monkhorst–Pack *k*-point grid. While this coarse grid is sufficient for accurately capturing the electronic structure of free-standing ice slabs (Figure S3), it is inadequate for Au-supported systems. In the latter, a denser *k*-point sampling is required to properly resolve the delocalized *s*-like states of Au around the Fermi level and to achieve convergence of the surface work function (Figure S4). Consequently, although structural relaxations for Au-supported systems were performed with a 1 × 1 × 1 grid, the final electronic structure and charge transfer calculations were carried out using a denser 4 × 4 × 1 grid.

Importantly, a dipole correction was applied in the (out-of-plane) *z*-direction of the simulation cells for two purposes: first, to obtain a plateau in the *xy*-averaged electrostatic potential to use as a vacuum reference for the calculation of the work function (WF), i.e., WF = Φ^vac^ – *E*_F_ with Φ^vac^ and *E*_F_ the vacuum potential and the Fermi level, respectively. And second, to *not* compensate for the depolarization field induced by the dipolar order of the ice slabs, as otherwise automatically done due to the periodic boundary conditions (PBC) implemented in plane-wave DFT codes.^20^

Fukui functions were computed throughout this study to assess and visualize the location of the electronic frontier orbitals participating in electronic transfer processes. The Fukui function helps to analyze the reactivity of a chemical system by revealing its redox center, i.e., the site where the system might lose or gain electrons during a reaction. The Fukui function *f*(**r**) is defined as the derivative of the electron density ρ­(**r**) with respect to the number of electrons *N* of the system

1 $$f\left(r\right)=\frac{\partial \rho \left(r\right)}{\partial N}$$

Depending on the type of electron exchange being studied, the Fukui function can take different forms. For a nucleophilic attack (electron gain), the positive Fukui function *f* ^+^(**r**) is defined as finite difference under addition of an extra electron to the system

2 $$f^{+}\left(r\right)=\rho_{N+1}\left(r\right)-\rho_{N}\left(r\right)$$

Vice versa, for an electrophilic attack (electron loss), the negative Fukui function *f* ^–^(**r**) is defined

3 $$f^{-}\left(r\right)=\rho_{N}\left(r\right)-\rho_{N-1}\left(r\right)$$

with ρ_*N*_(**r**), ρ_*N*+1_(**r**) and ρ_*N*–1_(**r**) the electron density of the neutral system and of the system with one extra electron added or removed, respectively. In practice, the Fukui functions have a dominant positive contribution associated with the active redox center, and minor oscillatory contributions due to the electronic response, i.e., the polarization, of the system.^29^

## Results

### Free-Standing Proton-Ordered Ice Slabs

For the unrelaxed free-standing ice slabs (from 1 to 6 bilayers), the projected density of states (PDOS) per atom type (H and O) and per bilayer are represented in Figures 1 and S5. A similar PDOS analysis for the relaxed systems is provided in Figure S6. When comparing the PDOS associated with the different bilayers, it is observed that the molecular orbitals are shifted in energy depending on the location of the bilayer within the ice slab. The orbital energies increase in a regular order, with the lowest (respectively highest) in energy corresponding to the bilayer at the edge of the ice slab with H pointing outward (reps. inward). These two specific bilayers are referred as bottom and top bilayers, respectively. Notably, the ice slab—which is insulating for a single bilayer (electronic band gap of *E*_g_ = 3.5 and 4.4 eV for the unrelaxed and relaxed systems, respectively)—becomes pseudometallic for two bilayers and more, i.e., the band gap of the overall system gets closed. These observations suggest an electron transfer from the HOMO of the water molecules in the top bilayer to the LUMO of the molecules in the bottom bilayer.

**Figure 1:**
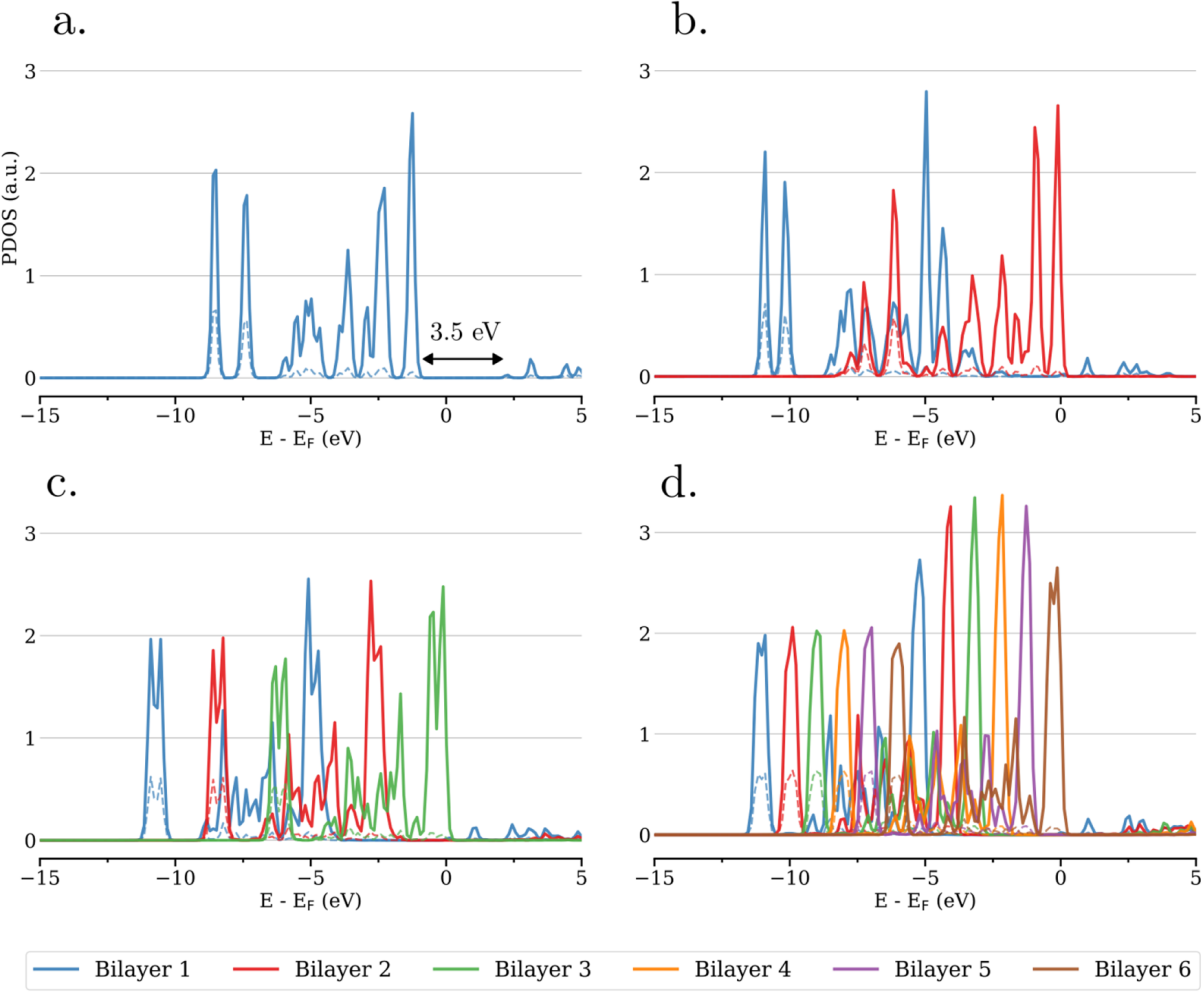
PDOS averaged per atom type and per bilayer for unrelaxed free-standing ice slabs. Panels (a–d) correspond to systems comprising 1, 2, 3, and 6 bilayers, respectively. Solid lines represent oxygen atoms, and dashed lines represent hydrogen atoms.

The occurrence of such an electron redistribution between the bilayers at opposite faces of the ice slab can be verified by integrating the charge per bilayer determined by Bader charge analysis. The obtained charge per bilayer is plotted as a function of the total number of bilayers in the slab in Figure 2a,2b for the unrelaxed and relaxed systems, respectively, with bilayers numbered starting with the bottom. For the unrelaxed case, the bottom bilayer presents a negative charge, which is increasing in magnitude with the number of bilayers in the slab. On the other hand, the top bilayer presents a positive counter charge in most cases, while the intermediate layers remain largely uncharged. This long-range electron transfer between the two ends of the ice slab leads to the formation of H_2_O^δ+^ and H_2_O^δ−^ species and therefore represents an autoredox process across the free-standing ice slab. Notably, this process is already observed for the case of only two free-standing proton-ordered bilayers.

**Figure 2:**
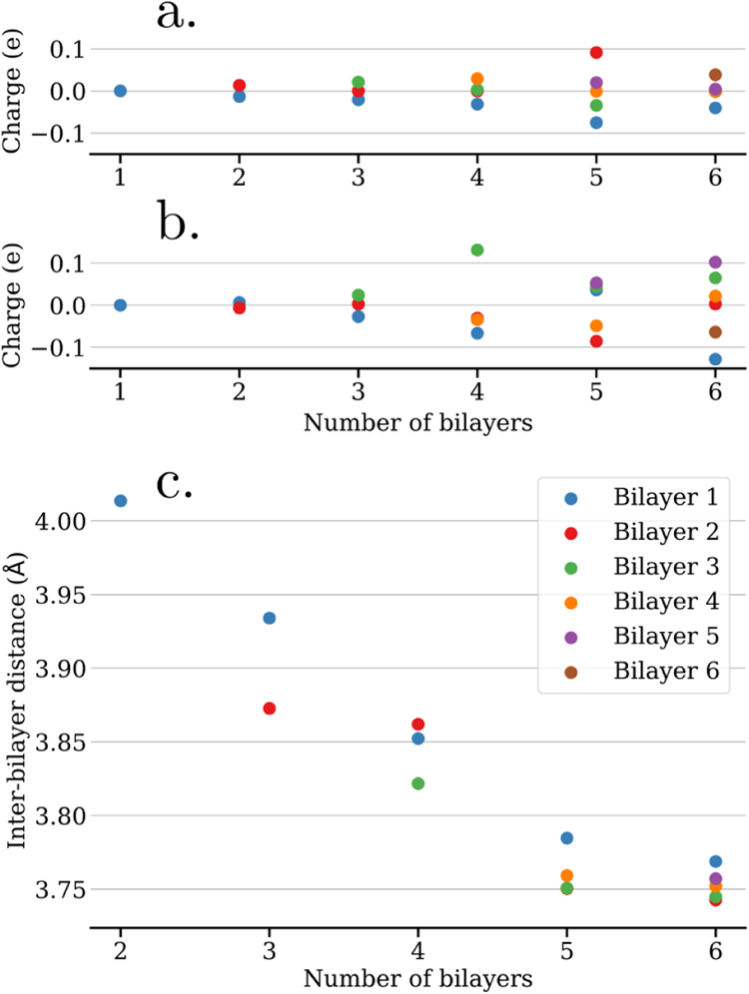
Bader charge per bilayer as a function of the total number of bilayers in (a) unrelaxed and (b) relaxed free-standing ice slabs. Panel (c) shows the interbilayer distances for the relaxed structures.

The system with 5 bilayers falls out of line. Here, the positive charge does not reside on the top bilayer but on bilayer 2, which is adjacent to the negatively charged bilayer 1, see Figure 2a. This behavior does not match the PDOS shown in Figure S5e, where the HOMO of bilayer 5 is dominating around the Fermi level and should thus represent the electron source for the negative charge of bilayer 1. We therefore consider the unexpected Bader charge of bilayer 2 to be due to an inaccurate charge partitioning, which is likely to happen for a system such as H_2_O with modest fluctuations in electron density.

Turning to the results for the relaxed ice slabs, the charge redistribution is more difficult to interpret due the superposition of several factors. Whereas the bottom bilayer generally retains a negative charge after structural relaxation, the positive counter charge generally does not anymore reside on the top bilayer. This picture is supported by the PDOS of the relaxed systems shown in Figure S6, where the shift of the orbital contributions from different bilayers does not follow a clear trend for the systems with more than 3 bilayers. Interestingly, the interbilayer distance is clearly decreasing when increasing the number of bilayers in the slab, as shown in Figure 2c, which is explained by an enhancement of the electron redistribution with increasing slab thickness, see Figure 2a,b. The enhanced opposite charging on both surfaces of the slab increases the electrostatic attraction between different bilayers and consequently decreases the interbilayer distances.

The redox-active frontier orbitals of the system can be best visualized by the means of the Fukui functions. As shown in Figures 3 and S7, the positive Fukui function *f* ^+^(**r**), corresponding to electron-accepting orbitals, is always localized on the bottom bilayer, while the negative Fukui function *f* ^–^(**r**), corresponding to electron-donating orbitals, is localized on the top bilayer of the free-standing ice slab. This result clearly demonstrates that, independent of the number of water bilayers, i.e., slab thickness, the redox-active orbitals of H-ordered ice slabs are localized on the two opposite slab surfaces.

**Figure 3:**
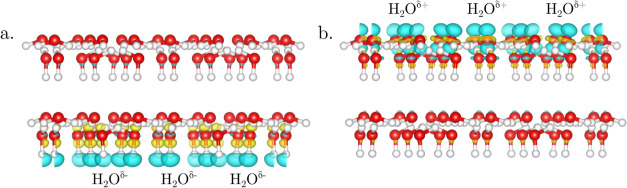
(a) Positive and (b) negative Fukui functions *f* ^+^(**r**) and *f* ^–^(**r**) of the relaxed free-standing ice slab comprising two water bilayers. The equi-density value used for the contour surface representation was 2 × 10^–6^ and 4 × 10^–6^ for *f* ^+^(**r**) and *f* ^–^(**r**), respectively.

### Supported Proton-Ordered Ice Slabs

The PDOS per bilayer for the unrelaxed systems of heteroepitaxial ice films supported on Au(111) substrate are shown in Figure 4 for both H-down (a–c) and H-up (d–f) orientations (with respect to the Au substrate). All PDOS for the Au(111)-supported unrelaxed and relaxed systems are provided in Figures S8–S11. For the supported ice slabs, we refer to the bottom and top bilayers as those that are closest and furthest from the Au substrate, respectively. Overall, the orbitals of different bilayers are shifted in energy following the order of how they are arranged within the ice slab, akin to observations made for free-standing ice slabs. For H-down orientation, the top bilayer’s orbitals are most positively shifted in energy, crossing the Fermi level with the respective HOMO. For H-down orientation, the top bilayer’s orbitals are most negatively shifted in energy, and the PDOS at the Fermi level is governed by the respective LUMO. Consequently, an electronic depletion of the HOMO of the top bilayer, with a resulting positive net charge, is expected for H-down configurations. *Vice versa*, an electronic accumulation in the LUMO of the top bilayer, with a resulting negative net charge, is expected for H-up configurations.

**Figure 4:**
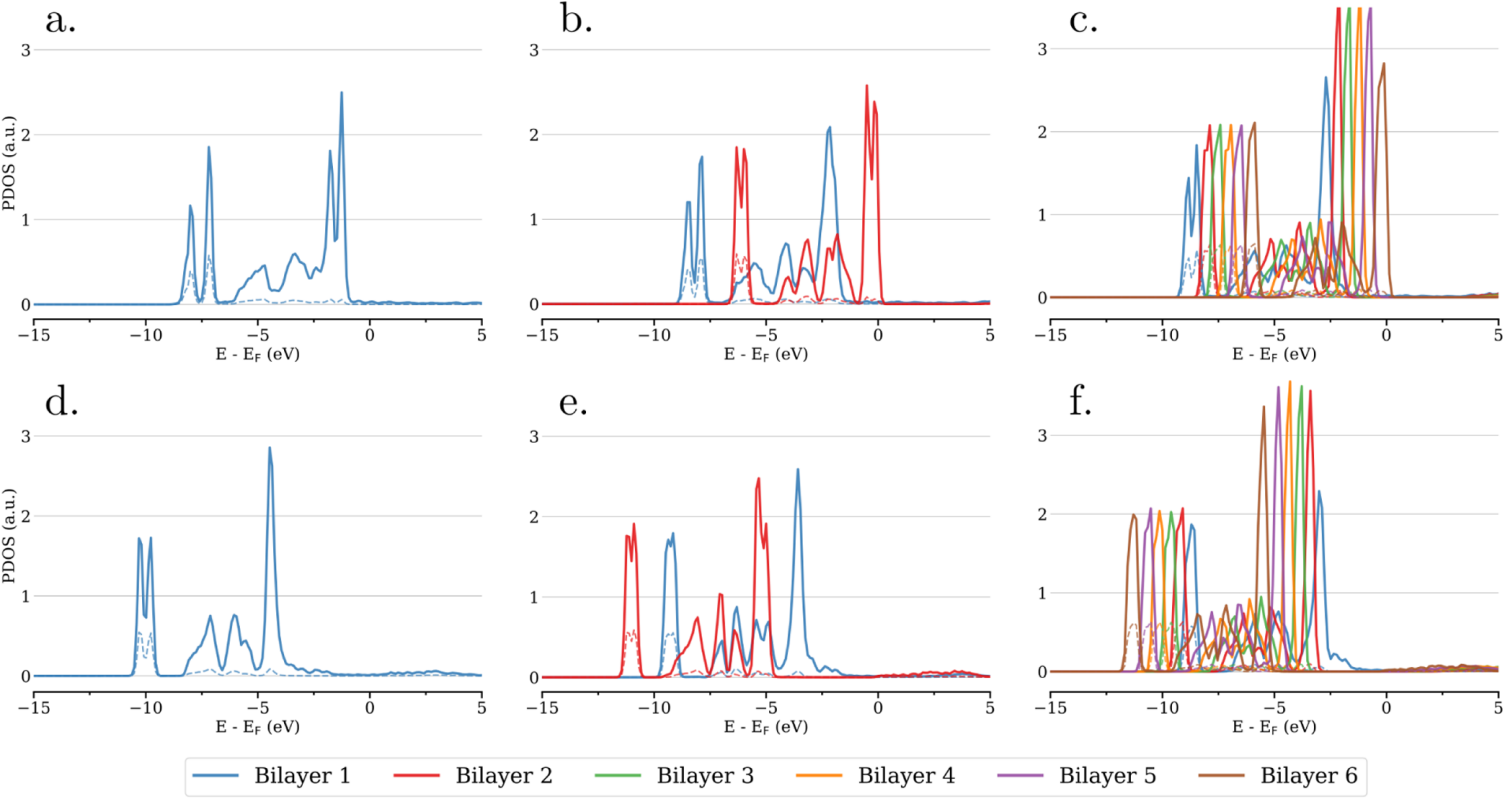
PDOS averaged per atom type and per bilayer for unrelaxed heteroepitaxial ice films supported on Au(111) substrate, whereby panels (a–c) correspond to 1, 2, and 6 bilayers in H-down configuration and (d–f) to the same in H-up configuration. Here, bilayer 1 always refers to first bilayer counted from the Au(111) surface regardless of H orientation.

This is indeed confirmed by the Bader charge analysis shown in Figure 5. For H-down configurations, the top bilayer always presents a positive charge, which is increasing with the number of bilayers in the slab, while for H-up configurations, the top bilayer always presents a negative charge, which is also increasing in magnitude with the number of bilayers. Again, top bilayer charging is already observed for the case of only two supported proton-ordered bilayers, but at a significantly larger magnitude than for the free-standing systems, cf. Figure 2a. Interestingly, in contrast to the free-standing ice slabs, the counter charge for the top bilayers of the supported systems is not provided by the respective bottom bilayers, which remain largely uncharged as shown in Figure 5. This observation demonstrates that an electron transfer is occurring between the Au substrate and the top water bilayer, from the HOMO of the top bilayer to the Fermi level of the electrode for H-down, and from the electrode Fermi level to the LUMO of the top bilayer for H-up orientation. This phenomenon is visualized by the Fukui functions for the Au(111)/ice system shown in Figure 6. Here, the negative Fukui function *f* ^–^(**r**), i.e., the electron-donating HOMO orbitals, of H-down oriented bilayers and the positive Fukui function *f* ^+^(**r**), i.e., the electron-accepting LUMO orbitals, of H-up oriented bilayers are both localized on the top bilayer. The fact that strongest electron transfer happens between the electrode surface and the *outermost* water bilayer is rather surprising, as one might expect the strongest interaction to occur with the bilayer in direct contact with the electrode. Similar observations of long-range electron redistribution at metal–solvent interfaces have been made in a previous work.^30^ As discussed in the following, this long-range redox process is mediated by the dipolar ordering of the intermediate solvent layers. It should be noted that the present considerations focus on the equilibrated system, while the kinetics of such long-range redox process will depend on the ratio between the strength of the electric field generated by the ferroelectric order and the distance over which the electron must tunnel. Strong local electric fields will shorten the tunneling distance and thus enhance the electron transfer kinetics.

**Figure 5:**
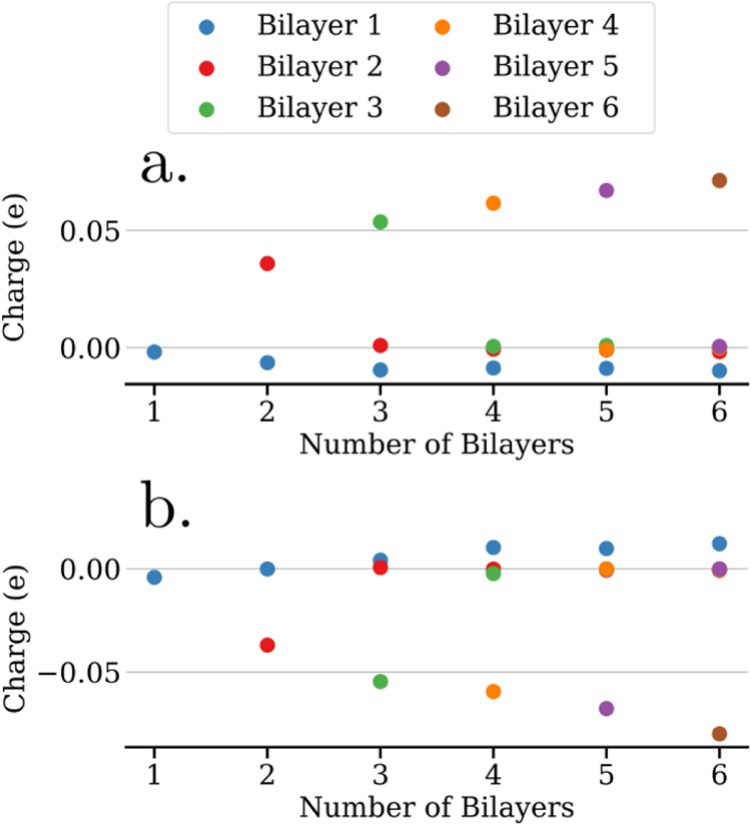
Bader charge per bilayer as a function of the total number of bilayers in relaxed heteroepitaxial ice films supported on Au(111) substrate, orientated in either (a) H-down or (b) H-up configuration.

**Figure 6:**
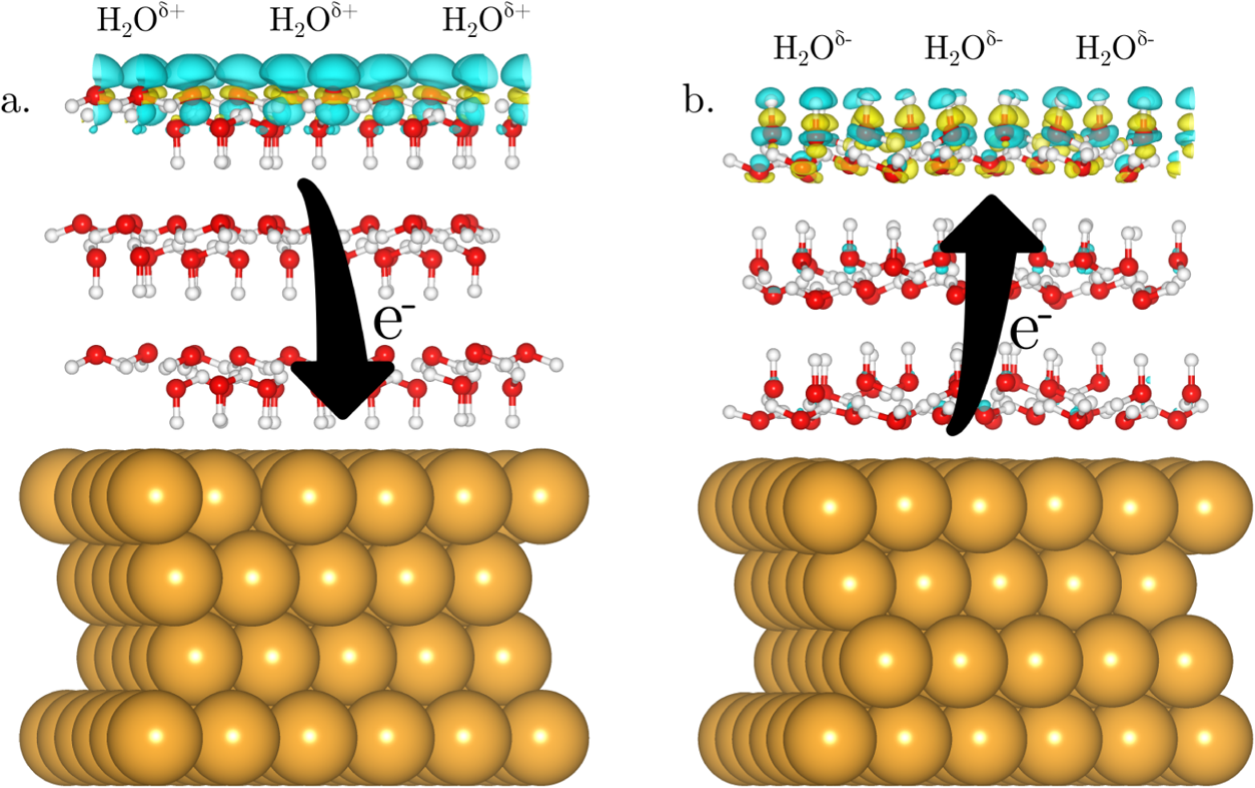
(a) Negative and (b) positive Fukui function, *f* ^–^(**r**) and *f* ^+^(**r**), computed for the relaxed heteroepitaxial ice film supported on Au(111) substrate containing three water bilayers in (a) H-down and (b) H-up configuration, respectively. The equi-density value used for the contour surface representation was 1 × 10^–6^ and 2 × 10^–6^ for *f* ^–^(**r**) and *f* ^+^(**r**), respectively.

Specific direct interactions between the Au surface and the closest bilayer are revealed by the minor negative (respectively positive) charges of the first bilayer for H-down (respectively H-up) configurations, cf. Figure 5. The first water layer becomes slightly metallized and thus participates in the electrode surface charging due to hybridization of molecular orbitals with the conduction band of the electrode, as described in a previous work.^31^

The computed WFs of the relaxed Au(111)/ice systems are plotted as a function of the number of ice bilayers in Figure 7. The computed WF for the bare Au(111) surface was found at 5.14 eV, which is close to the experimental value of 5.33 eV.^32^ The WF value of the bare Au(111) surface reflects the strength of the intrinsic surface dipole denoted as *D*_m_ in Figure 7, which is induced by spillover of the electron density tail into the vacuum region and points down toward the electrode surface. Adding water bilayers in H-down orientation increases the effective WF by several eV, while H-up oriented bilayers result in a decrease of the WF. As visualized in Figure 7, this effect is due to the oriented dipoles *D*_ori._^w^ of the added water bilayers, which either increase or decrease the overall surface dipole depending on the bilayer orientation. Surprisingly, the effective WF is not continuously increasing (respectively decreasing) while adding H-down (respectively H-up) bilayers onto the system, but quickly reaches a saturation limit. Work function saturation has also been observed for water adlayers on Pt(111) by Tripkovic et al.,^33^ however, without further investigating the origin of this effect. Here, we find that WF saturation is reached for a Au(111) surface covered by two or more ice bilayers. Saturation is caused by the long-range electron transfer between the electrode surface and the top water bilayer, as established from Bader analysis, which creates an additional charge transfer (c.t.) dipole *D*_c.t._ pointing in opposite direction than the oriented molecular water dipoles and canceling the added *D*_ori._^w^ per bilayer. This finding matches the PDOS results where two H-ordered bilayers were sufficient to cross the Fermi level with either HOMO or LUMO of the top bilayer (for H-down or H-up orientation, respectively) and trigger electron transfer to/from the electrode. The formation of H_2_O^δ+^ or H_2_O^δ−^ at the top layer of the ice slab thus induces a new dipole perpendicular to the surface that compensates the orientational dipoles of bilayers and limits the WF increase/decrease.

**Figure 7:**
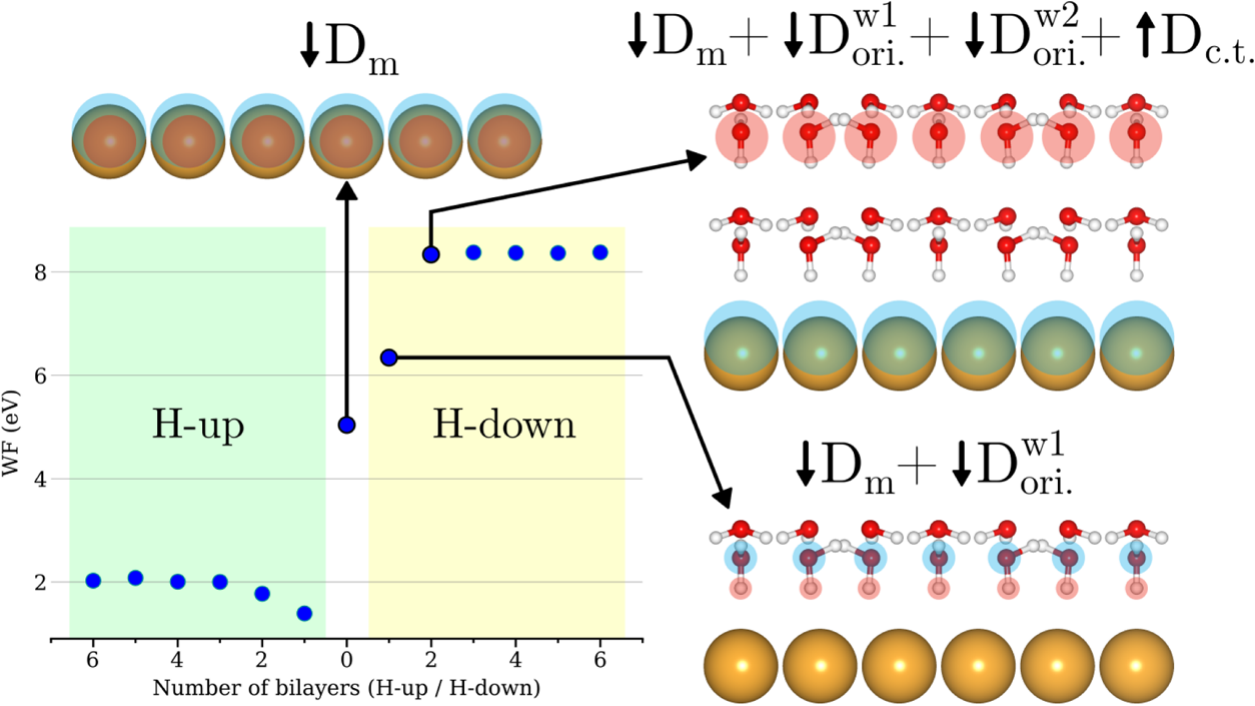
Computed effective work function (WF) of the relaxed Au(111)/ice systems as a function of the number of ice bilayers (H-up and H-down). The schematic representations visualize the different dipolar contributions to the WF, where red and blue spheres indicate the respective positive and negative charges.

## Discussion

The depolarization field in ferroelectrics originates from the uncompensated bound dipolar charges at the material’s surfaces due to the dielectric discontinuity. If not adequately screened by free charge carriers, the depolarization field, which opposes the direction of the internal ferroelectric polarization, can significantly destabilize the ferroelectric state.^34^ Regarding the proton-ordered phase of ice XI, ionic impurities such as KOH or NaOH can provide the required free charges to compensate the depolarization field and stabilize the ferroelectric state.^11,13–15^ Interestingly, Parkkinen et al.^20^ suggested that compensation charges can also be generated intrinsically in the form of H_3_O^+^ and OH^–^ species due to field-induced dissociation of water molecules by the depolarization field. In our simulations, we did not observe such spontaneous dissociation of water molecules. Nevertheless, the established formation of partially ionized water molecules (H_2_O^δ+^ and H_2_O^δ−^) due to long-range autoredox across ferroelectric ice slabs can be regarded as a first step to activate and destabilize water molecules toward subsequent dissociation. The autoredox process proposed here is thus complementary to the dissociation mechanism suggested by Parkkinen et al. Moreover, our results for the Au-supported ice systems precisely confirm the electron and hole injection mechanisms proposed by Nie et al.^23^ and Sugimoto et al.,^24^ respectively, as a result from the bending of the electronic band structure of the ice film by the depolarization field. We here showed that Au-supported H-down (respectively H-up) oriented ice XI films naturally transfer electrons from the top water bilayer to the substrate electrode (respectively from the electrode to the top water bilayer), leading to the formation of H_2_O^δ+^ (respectively H_2_O^δ−^) species at the surface of the water film. Notably, our results show that the injected holes or electrons are localized on the outermost water bilayer, leaving the intermediate layers unaffected, regardless of the ice film thickness. These findings suggest a close correspondence between charge injection mechanisms of supported ferroelectric ice films and field-induced water dissociation in free-standing ferroelectric ice slabs.

To better visualize the origin of the energy shift of the water bilayer orbitals observed in Figures 1 and 4, the in-plane averaged electrostatic potential of the free-standing ice slabs is plotted as a function of the out-of-plane *z*-coordinate in Figure 8a. It is seen that each proton-ordered water bilayers contributes an electrostatic potential step that shifts the bilayer’s molecular orbitals in energy. We find here that a single oriented water bilayer produces a potential step of ΔΦ_*z*_^bl^ = 4.2 eV, which corresponds to an average out-of-plane dipole moment of about *p*_*z*_ = 1.0 D per water molecule (see calculation in the SI). This value is smaller than the total dipole moment of a water molecule of |*p⃗*| = 1.85 D,^35^ which is reasonable since not all of the molecular dipoles within an ordered water bilayer are pointing out of plane. A different bilayer’s orientation (leading to a different *p*_*z*_) and density (or molecular occupancy area) would have resulted in a different potential step (see Figure S12).

**Figure 8:**
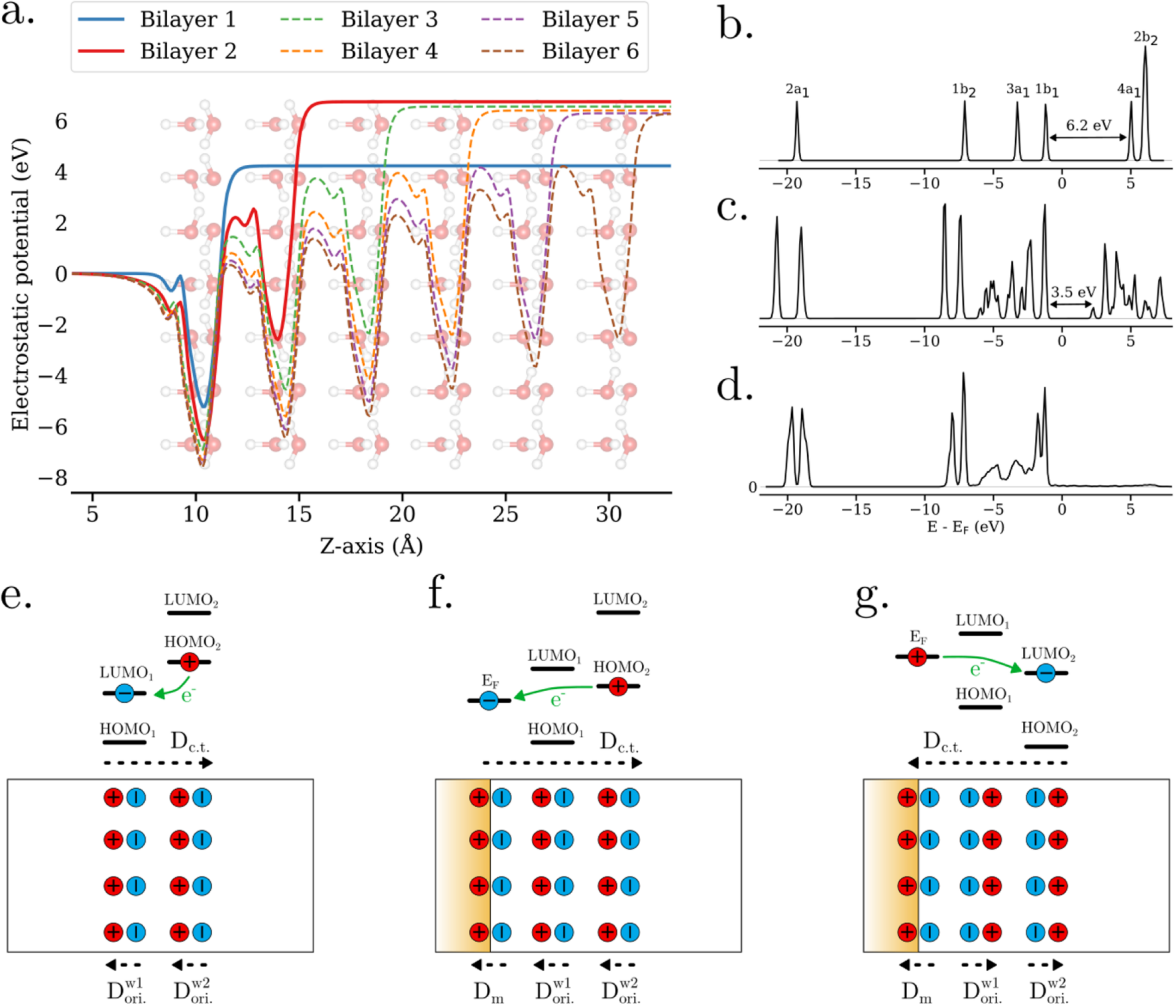
(a) Electrostatic potential profiles of the simulation cells containing free-standing ice slabs, averaged in-plane and plotted along the out-of-plane *z*-axis. (b) Computed DOS of a single water molecule. The peak labels correspond to the known molecular orbitals of water. (c) Computed DOS of a single free-standing ice bilayer. (d) Computed DOS (containing only O and H contributions) of a single Au(111)-supported ice bilayer. Panels (e–g) represent the interplay between the oriented (ori.) dipoles *D*_ori._^wi^ and the charge transfer (c.t.) dipoles *D*_c.t._, and the origin of the c.t. dipoles induced by the long-range electron transfer in (e) free-standing ice, (f) H-down supported ice, and (g) H-up supported ice.

It is interesting to compare the value of ΔΦ_*z*_^bl^ = 4.2 eV for the per-bilayer potential step with the effective HOMO–LUMO gap of a water bilayer. For a single water molecule, we obtained a value of *E*_*g*_^mol^ = 6.2 eV at the DFT-PBE level (cf. Figure 8b), consistent with other reported values at the DFT-LDA/GGA level.^36,37^ Notably, the computed HOMO–LUMO gap of an entire water bilayer is decreased to a value of *E*_g_^bl^ = 3.5 eV, as shown in Figure 8c. This decrease originates from the spatial variations of the electrostatic potential due to different orientations of molecular dipoles within the bilayer, which result in a distribution of molecular HOMO and LUMO energies and an effectively smaller overall gap. Based on these values, the *critical* number of oriented water bilayers required to induce an autoredox process can be estimated, *N*_cr_^bl^ = *E*_g_^bl^/ΔΦ_*z*_^bl^ = 3.5/4.2 = 0.8, meaning that two water bilayers are already sufficient to cause a crossing between the HOMO of the one and the LUMO of the other and thus induce transfer of electrons (cf. Figure 8e). This result is matching our findings discussed above, where the autoredox process was found to occur on free-standing and supported ferroelectric ice slabs containing two bilayers or more, cf. Figures 1b and 2a,b. This result is also reflected in Figure 8a, where two water bilayers are seen to produce a vertical potential step of ΔΦ_*z*_ = 6.8 eV while a value of ∼8.4 eV would be expected by simply adding the individual bilayers’ potential steps. For 3, 4, 5, and 6 bilayers, the overall potential step remained approximately equal as for the two bilayer system, which, as discussed before, is due to the formation of a charge transfer dipole *D*_c.t._ that compensates for the cumulated potential steps of added bilayers, cf. Figures 7 and 8e–g. However, it is important to note that these results strongly depend on the effective HOMO–LUMO gap obtained from the computational method used. As discussed later on, the here employed GGA-DFT method generally underestimates the effective gap, for which reason the estimated critical number of two ice bilayers sufficient for triggering autoredox must be taken with some care. Nonetheless, even using a twice-as-large value for the effective gap would yield the same critical number of only two ice bilayers.

The present findings suggest the compensation of the depolarization field, and thus the stabilization of ferroelectric ordering in ice films, by internal, or substrate-mediated redox processes. There exists a strong coupling between the alignment of water dipoles and the activation of water molecules by electronic charge extraction from (or injection into) the molecular HOMO (or LUMO) states. This finding is particularly interesting when transferred to the context of electrocatalytic reactions where the activation of water molecules toward dissociation often corresponds to the rate-determining step. A well-known example for this is the hydrogen evolution reaction (HER) in alkaline media, for which a detailed understanding of the interrelated effects of electrode material, applied potential, electrolyte pH, as well as cation type and concentration is still lacking.^38–41^ The kinetics of the HER in alkaline electrolytes are controlled by the first electron transfer step (Volmer step), H_2_O + e^–^ + * → *H + OH^–^ (here, * denotes a free surface adsorption site), which itself is governed by the dissociation of the water molecule.^38^ Nonetheless, it is still unclear by what detailed mechanism the water molecule is dissociated and the adsorbed hydrogen *H is formed under the influence of negative potentials applied for alkaline HER. From AIMD simulations, it has been found that at negative potentials (with respect to the potential of zero charge), the first interfacial water layer at the electrode surface is partially H-down oriented.^42^ Because this dipolar ordering is caused by the action of the electric field in the electrochemical double layer (EDL), it is understood that the *average* dipole moment of the first water layer, i.e., the average degree of proton ordering, must remain significantly weaker than the dipole moment of a fully oriented bilayer of ferroelectric ice. However, the question is raised whether *local fluctuations* of the dipolar alignment of water molecules in the EDL can become sufficiently strong to trigger electronic redox processes similar to the present observations for ferroelectric ice films. In such a scenario, electron tunneling could become feasible from the HOMO of water molecules in the *second* layer to the electrode surface leading to the formation of H_2_O^+^ species. By reacting with neighboring water molecules of the first layer, the activated H_2_O^+^ could dissociate into mobile protons and ^•^OH radicals through H_2_O^+^ + H_2_O → H_3_O^+^ + ^•^OH. The mobile protons of H_3_O^+^ can migrate to free adsorption sites at the electrode surface and get discharged to form *H. The ^•^OH radicals could either get stabilized by electron (back) transfer from the negatively charged electrode surface via ^•^OH + e^–^ → OH^–^ or react with a neighboring radical via ^•^OH + ^•^OH → H_2_O_2_. Whether, and to what extent, such a mechanism of sequential electron tunneling and proton shuttling can contribute to the alkaline Volmer step will be an interesting question for subsequent studies.

Based on our findings, we propose here an estimation of the number of water molecules in (instantaneous) dipolar alignment, *N*_cr_^liq^, required to trigger *local* redox activation of water molecules in the context of alkaline HER (i.e., liquid water), using the relation *N*_cr_^liq^ = *E*_g_^liq^/ΔΦ^liq^. This requires the knowledge of the effective band gap of liquid water, *E*_g_^liq^, and an estimation of the local potential variation, ΔΦ^liq^, induced per molecular water dipole. As highlighted in various studies,^36,43–45^ the Kohn–Sham (KS) HOMO–LUMO gap of an isolated water molecule with a value of approximately 6 eV obtained from LDA or GGA functionals, is subject to two key limitations: (1) LDA and GGA functionals typically underestimate the gap due to enhanced delocalization, and thus overlap, of KS orbitals, and (2) there is a conceptual difference between the KS gap and the fundamental gap for electronic red/ox processes. The fundamental gap of liquid water has been the object of numerous experimental and computational works. Recently, Bischoff et al.^45^ reviewed various experimental studies to infer the fundamental gap of liquid water and reached a consensus for the experimental value of *E*_fg_^exp^ = 9.0 ± 0.2 eV. The authors computed the gap with different state-of-the-art many-body perturbation theory methods based on the GW approximation, as well as advanced hybrid functionals, and concluded a computational value of *E*_fg_^comp^ = 9.1 ± 0.1 eV, in excellent agreement with the experimental result.^45^ A comparison between the fundamental gap value for liquid water and the one of ice (determined experimentally and computationally),^45^ indicates that the fundamental gap of water is only weakly dependent on the crystal structure and temperature, meaning that a value of ∼9 eV can be employed in our estimation of the critical number of spontaneously aligned water molecules, *N*_cr_^liq^.

To estimate the local potential fluctuation, ΔΦ^liq^, induced by a molecular water dipole, we first computed the electrostatic potential surrounding a single water molecule in vacuum (Figure 9a). As expected from the positive/negative polarization of H/O atoms, the local potential increases from below the water molecule to above it. Sakong et al. showed by AIMD simulations that O–H distances between neighboring water molecules in liquid water are typically approximately 1.75 Å.^7^ Using this O–H distance, we observe that two molecules localized below and above a single water molecule can experience a potential step of ∼2.5 eV. To better represent realistic conditions, we then evaluated the electrostatic potential experienced by water molecules in a liquid water bulk environment. A system consisting of 32 water molecules, previously equilibrated by Tal et al. using AIMD simulations with the PBE functional,^43,46^ was employed. To evaluate the electrostatic potential experienced by the water molecules, each water molecule was individually removed from the simulation cell, and the potential was computed at the center of mass (COM) of the removed molecule. Figure 9b shows a representative two-dimensional (2D) potential map in a plane intersecting a water molecule, while Figure 9c presents the corresponding statistical distribution of the COM’s potential. The spread (i.e., fluctuations) of the values of the potential experienced by a water molecule in liquid water, ΔΦ^liq^, is found to be approximately 2.5 eV, which is very similar to the case of the isolated water molecule. Thus, together with the value of about 9 eV for the fundamental gap, the critical number of spontaneously aligned water molecules is evaluated to *N*_cr_^liq^ = *E*_g_^liq^/ΔΦ^liq^ = 3.6, meaning that four water molecules in serial dipolar alignment are required to trigger autoredox processes in liquid water. Interestingly, the rarity of such aligned configurations may be consistent with the infrequent nature of autoionization events in liquid water, which are known to occur only on time scales of several hours.^47^

**Figure 9:**
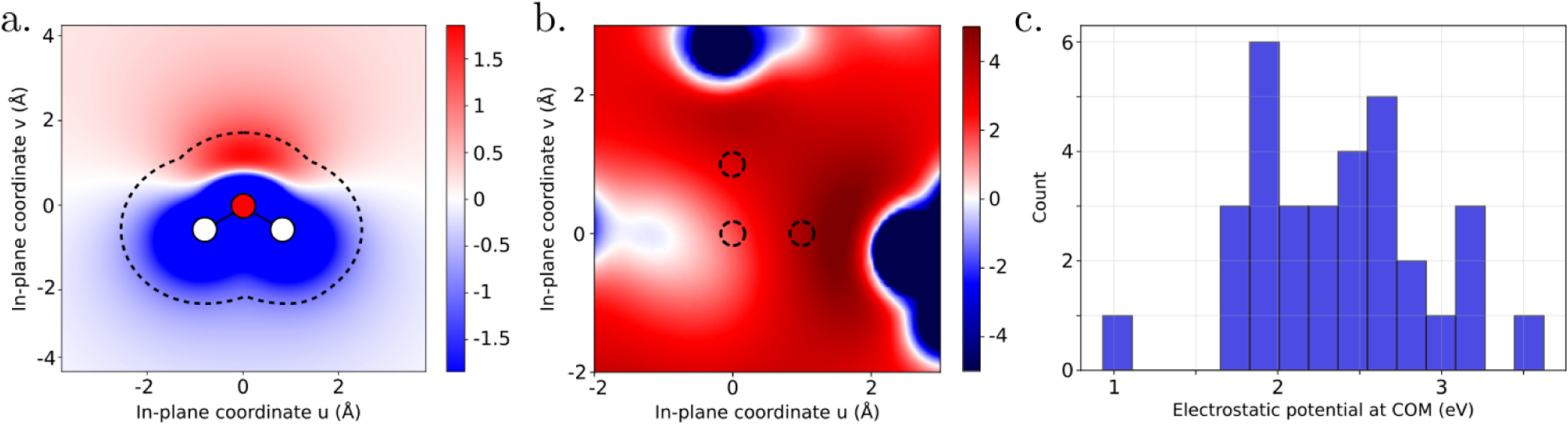
Electrostatic potential (in eV) in the molecular plane of a selected water molecule: (a) an isolated water molecule in vacuum and (b) a liquid water box containing 32 molecules equilibrated by AIMD from Tal et al.,^43,46^ with the selected molecule removed to evaluate the potential at its COM. In (a), the dotted contour indicates the typical spatial extent of neighboring molecules, based on an intermolecular O–H distance of 1.75 Å.^7^ In (b), the dotted circles mark the original location of the removed molecule. The in-plane coordinates *u* and *v* are defined within the molecular plane. (c) Distribution of electrostatic potentials evaluated at the COM of each of the 32 water molecules in the liquid box.

At an interface with a metal electrode, e.g., under HER conditions, this requirement is further relaxed, because the effective gap *E*_g_ to be overcome for redox corresponds to the difference between the molecular HOMO (or LUMO) of water and the Fermi level of the electrode (cf. Figure 8f), instead of the full HOMO–LUMO gap of water. For water oxidation, the effective gap between the molecular HOMO and metal Fermi level is decisive, while for water reduction, the effective gap from the metal Fermi level to the molecular LUMO matters. As shown in our results, cf. Figure S13, the Fermi level of the Au(111) electrode is located well within the band gap of the interfacial water bilayer. This effective redox gap is expected to depend on the nature of the metal surface and may be reduced for metals with higher work functions, such as Pt and Ru, which exhibit stronger chemisorption properties than Au and are known to facilitate water dissociation.^48^ It is also worth noting that under water oxidation conditions, such as those relevant to the oxygen evolution reaction (OER), the surface of metals, such as Ru, get oxidized, and the additional surface dipole due to the surface oxide layer effectively increases the surface work function. This brings the Fermi level of the electrode closer to the water molecular HOMO, thereby facilitating the activation of interfacial water molecules for oxidation. As such, both the metal identity and the degree of surface oxidation or coverage are expected to influence the value of *N*_cr_ by modulating the surface work function and thus the effective gap *E*_g_. Local potential variations of about 2.5 eV induced by single water dipoles aligned perpendicular to the interface thus appear sufficient for mediating electronic redox transfer between the electrode surface and second-row water molecules. Since out-of-plane dipole alignment becomes increasingly favored at enhanced surface charging, this process is expected to be strongly dependent on the applied electrode potential. It will be an interesting question for subsequent works to verify whether such dipole-fluctuation-induced redox activation of water molecules is a feasible pathway toward water dissociation under alkaline HER conditions.

Before concluding, we briefly discuss some similarities and differences between the behavior of ferroelectric and ferromagnetic materials. Ferromagnetism and ferroelectricity have very different physical origins but are governed by similar physical equations in terms of dipole–dipole and dipole–field interactions, leading to certain similarities in phenomena (ferro-to-para transition with increasing temperature, hysteresis, etc.). Nevertheless, the present work allows understanding a big limitation of ferroelectrics, originating from the unavoidable coupling between ferroelectric ordering and HOMO–LUMO alignment. For high enough internal electric field, i.e., sufficient degree of ferroelectric ordering, electrons will be transferred by tunneling, creating a local redox process that limits the maximum self-field. In the case of water, this effect is critical and restricts the extent of pure H-up or H-down organization by producing charged species. This suggests that ferroelectric ice XI would be associated with ionized H_2_O to stabilize its structure and limit the self-redox process. Likewise, the stabilization of ice XI by KOH doping is due to suppression of self-ionization by cancellation of the internal electric field. The same stabilization mechanism can be obtained by electron-beam or ultraviolet (UV) irradiation that are known to ionize water. On the contrary, in ferro*magnetic* materials, no such limitation by coupling between ordering and redox activation exists, allowing for ferromagnetic ordering at macroscopic scales, as, e.g., in technical permanent magnets.

Similar to ferromagnetic materials, however, another way to reduce the global internal electric field in ferroelectric ice films could be the formation of H-up and H-down *domains* with opposed electric orientations. The domain size would result from the balance between the cost of domain walls and the gain of reducing the average electric field. In the case of ice, the formation of large domains will also be prevented by the electron transfer and self-redox effects studied in this article. The possible existence of (small-scale) electric domains still needs to be investigated but could also lead to interesting organization structures at the interface, similar to what was previously computed for the ice bilayer.^49^

## Conclusions

In summary, we here studied the coupling between dipolar alignment and electronic redox phenomena in both free-standing and metal–supported ice films. Performing DFT simulations, it was found that about two ferroelectrically ordered bilayers of ice were already sufficient to cause HOMO–LUMO crossing and induce transfer of electrons. For free-standing ice films, the electronic redox involved the most distant bilayers, causing opposite charging of the ice-film surfaces. The resulting electric field cancels the internal depolarization field of the aligned dipole layers and thus stabilizes the ferroelectric ordering. For Au(111)-supported ice films, only a single water bilayer in either H-down or H-up orientation was found to be stable, while for two and more water bilayers a crossing between the metal Fermi level and the HOMO or LUMO states of the *outermost* water bilayer occurred. For these systems, redox activation of water molecules was thus observed at the largest distance from the metal surface, and not, as intuitively expected, at the direct interface with the substrate. These findings suggest that, within the limitations of static DFT calculations using the GGA-PBE functional, including more than a single ice-like water layer may push the system beyond the electrochemical stability window of water, leading to charge rearrangements and band alignments. On the other hand, the dipole-mediated “long-range” activation of water molecules might have real implications for electrocatalytic reactions at strongly charged electrode–electrolyte interfaces, such as electrochemical hydrogen evolution under alkaline conditions. In this context, it is suggested as a hypothesis that *local fluctuations* in the alignment of water dipoles can become sufficiently strong for triggering electronic redox transfer between the metal electrode and water molecules *from the second layer*. Such redox-activated molecules are prone to subsequent dissociation, providing mobile protons for the critical Volmer step. It will be the subject of subsequent studies to verify whether such a mechanism is feasible under alkaline HER conditions.

## Supplementary Material



## Notes

Initial geometries of free-standing and Au(111)-supported ice slabs (Figures S1–S2); *k*-point convergence tests for the total density of states and work function (Figures S3–S4); projected density of states for free-standing and supported ice slabs in various configurations (Figures S5–S6, S8–S11, S13); Fukui functions for relaxed ice slabs (Figure S7); a brief derivation of the average out-of-plane dipole moment per water molecule, and the resulting potential step as a function of dipole moment and occupancy area (Figure S12) (PDF)
