# Supplementary material for: Long-Range Redox and Water Activation at Metal–Water Interfaces with Ferroelectric Ordering
Source: J Chem Theory Comput. 2025 Jul 24;21(15):7636–47. doi: 10.1021/acs.jctc.5c00814 (PMC12355686; doi:10.1021/acs.jctc.5c00814)
Supplement: Supplementary file 1 [file ct5c00814_si_001.pdf]

Supporting Information for Long-Range Redox and Water Activation at Metal–Water Interfaces with Ferroelectric Ordering

Arthur Hagopian,*,† Jean-Sébastien Filhol,‡ and Tobias Binninger¶

†*Leiden Institute of Chemistry, Leiden University, Leiden 2333CC, The Netherlands*

‡*ICGM, CNRS, University of Montpellier, Montpellier, France*

¶*Theory and Computation of Energy Materials (IET-3), Institute of Energy Technologies, Forschungszentrum Jülich GmbH, 52425 Jülich, Germany*

E-mail: arth.hagopian@gmail.com

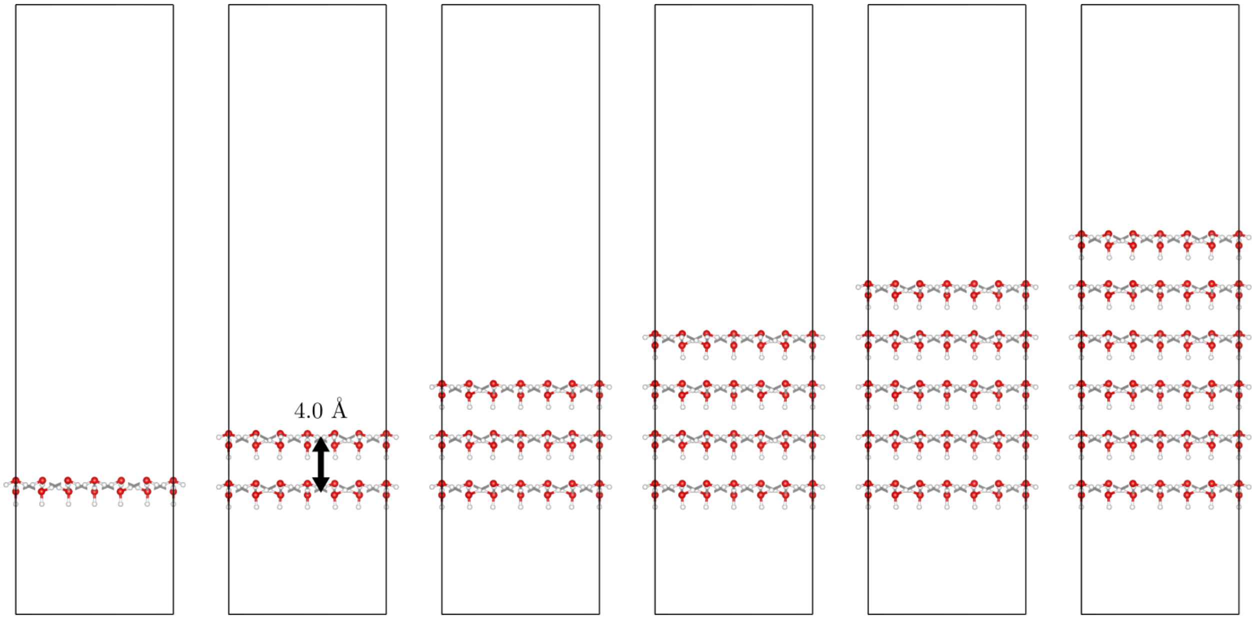

Figure S1: Initial geometries of the free-standing ice slabs. The inter-bilayer distance of 4.0 Å was obtained by optimazing the geometry of the 2-bilayers slab.

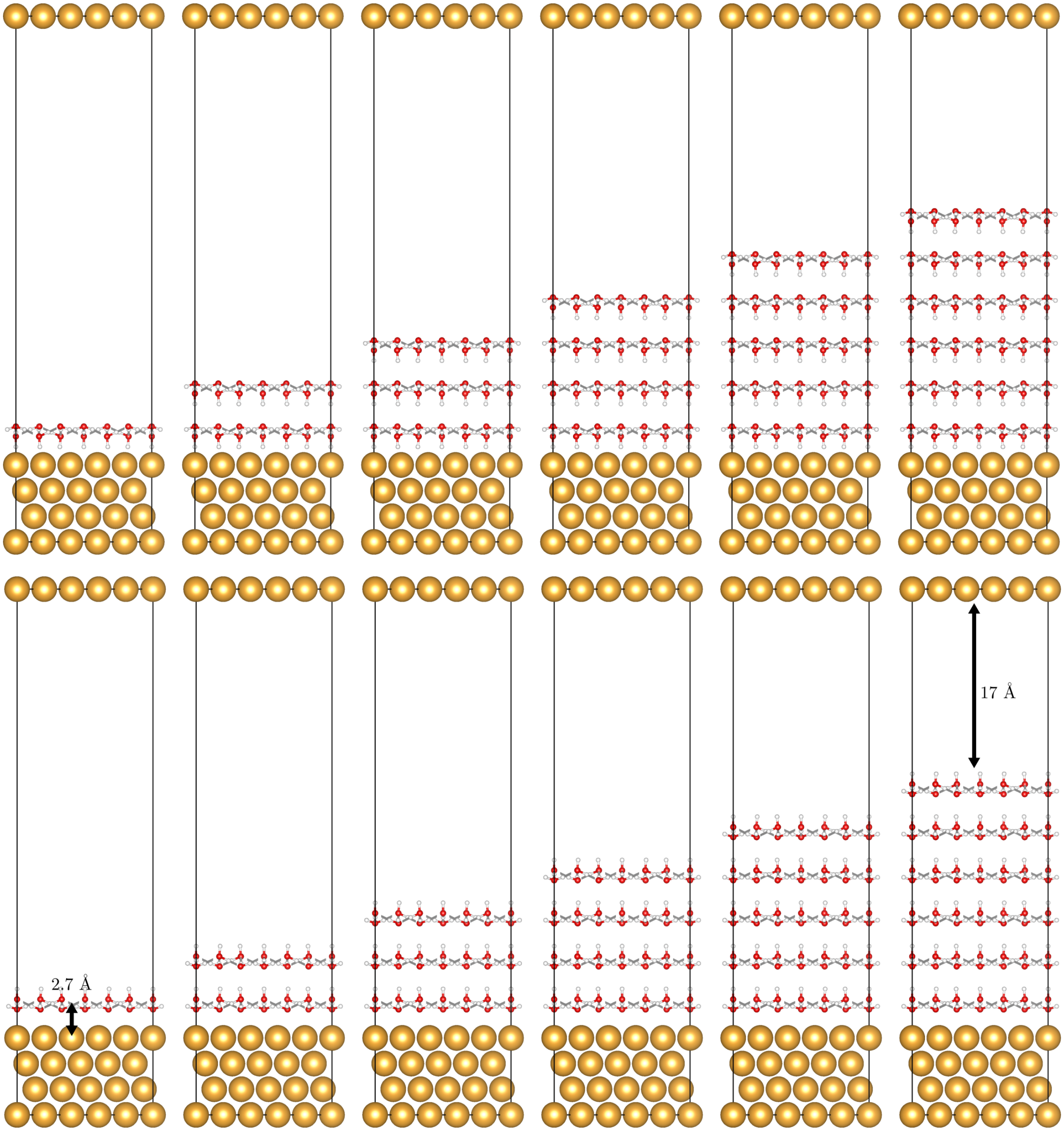

Figure S2: Initial geometries of the ice slabs supported on the Au(111) surface. The ice slabs were placed at a distance of 2.7 Å from the surface. The vacuum region of 42 Å ensures that at least 17 Å remains when the largest ice slab (6 water bilayers) is inserted in the cell. H-down and H-up orientated ice slab configurations are represented on the top and bottom part of the figure, respectively.

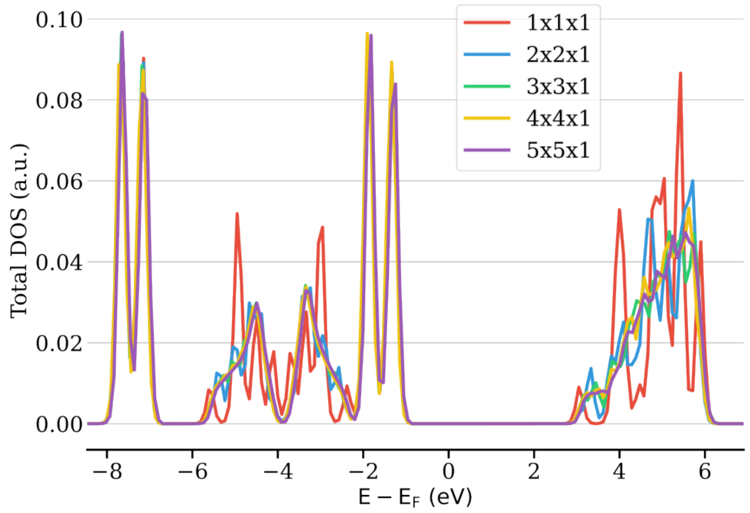

Figure S3: Total density of states of the relaxed free-standing single water bilayer, as a function of the k-point grid.

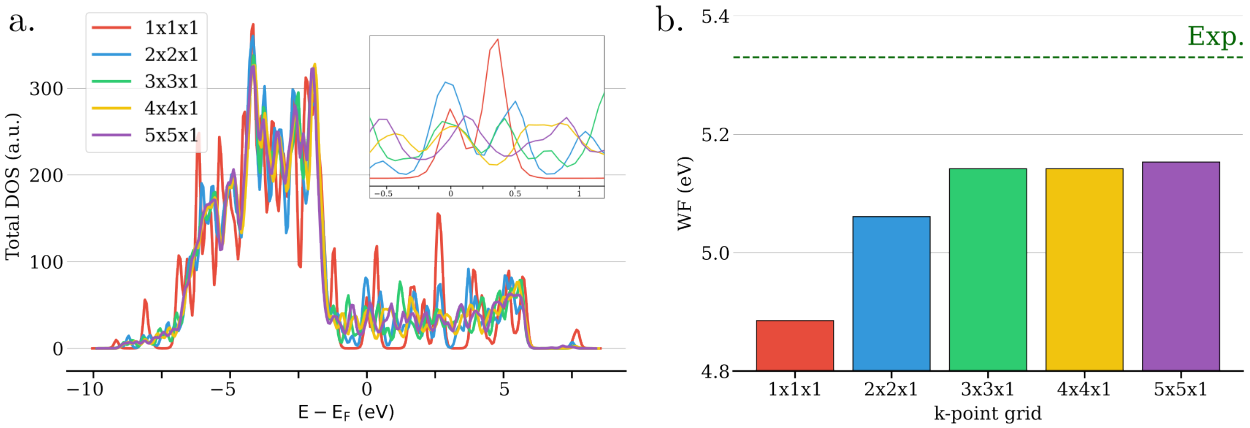

Figure S4: (a) Total density of states and (b) work function of Au(111) bare surface, as a function of the k-point grid.

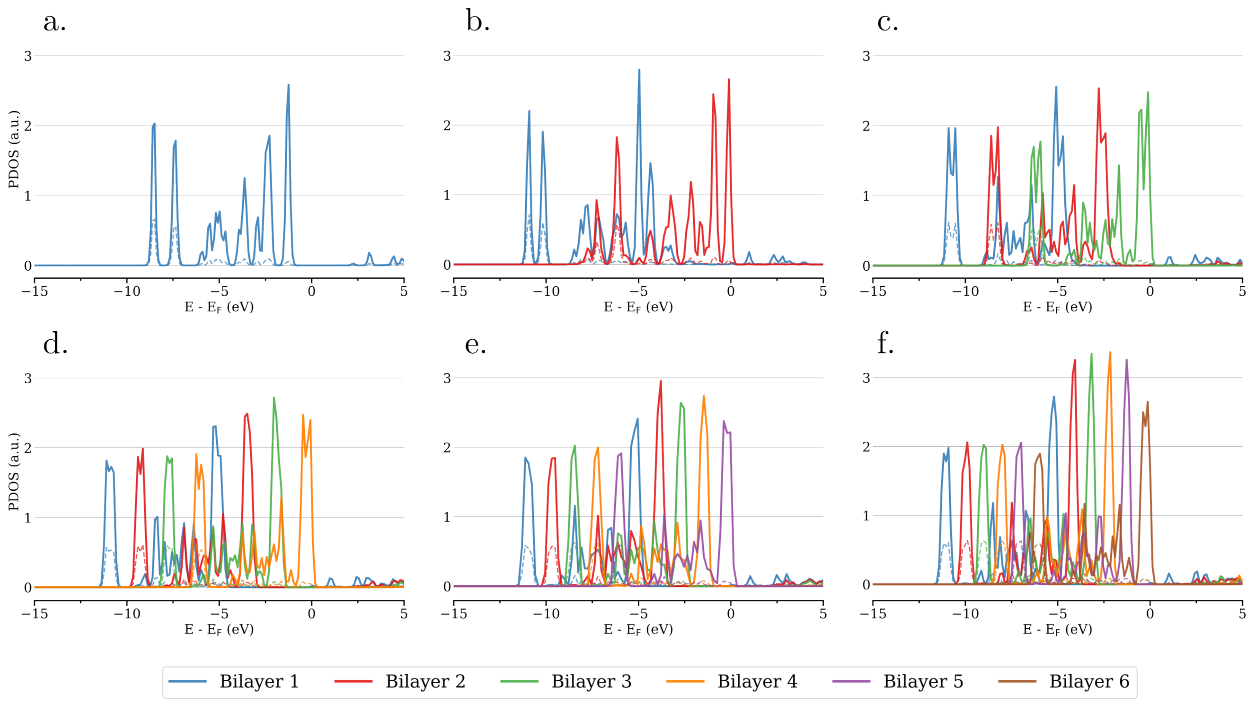

Figure S5: PDOS averaged per atom type and per bilayer for unrelaxed free-standing ice slabs. Panels (a–f) correspond to systems comprising 1, 2, 3, 4, 5 and 6 bilayers, respectively. Solid lines represent oxygen atoms, and dashed lines represent hydrogen atoms.

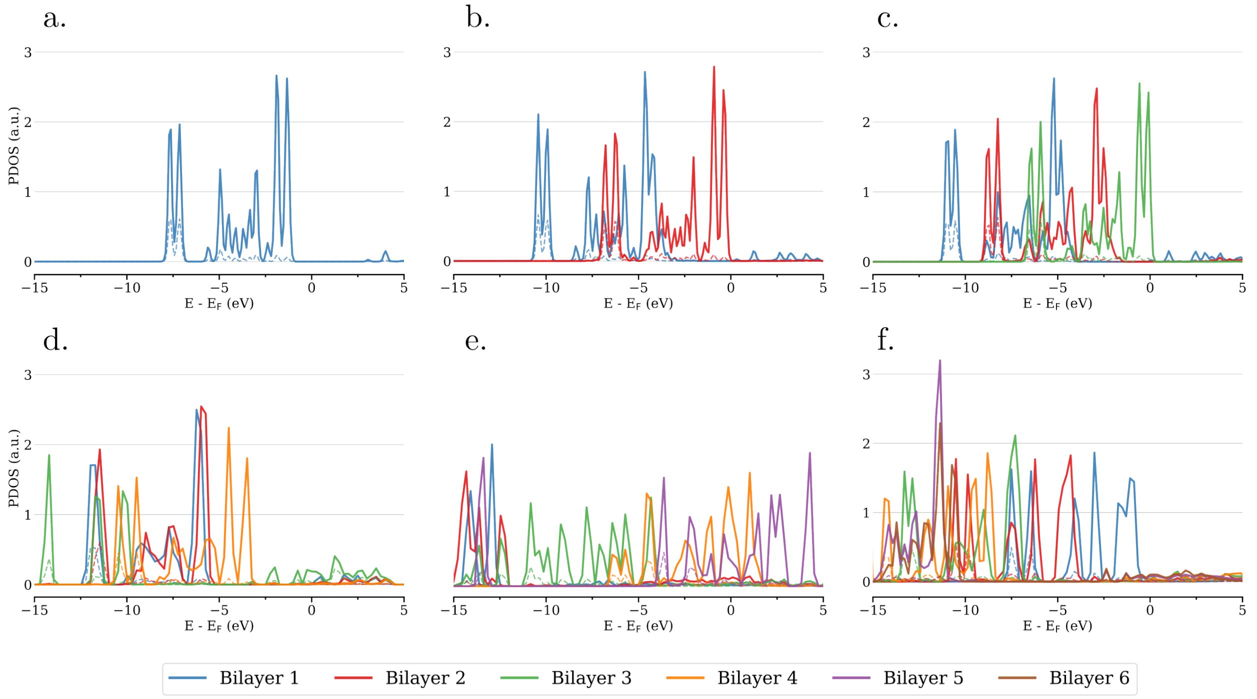

Figure S6: PDOS averaged per atom type and per bilayer for relaxed free-standing ice slabs. Panels (a–f) correspond to systems comprising 1, 2, 3, 4, 5 and 6 bilayers, respectively. Solid lines represent oxygen atoms, and dashed lines represent hydrogen atoms.

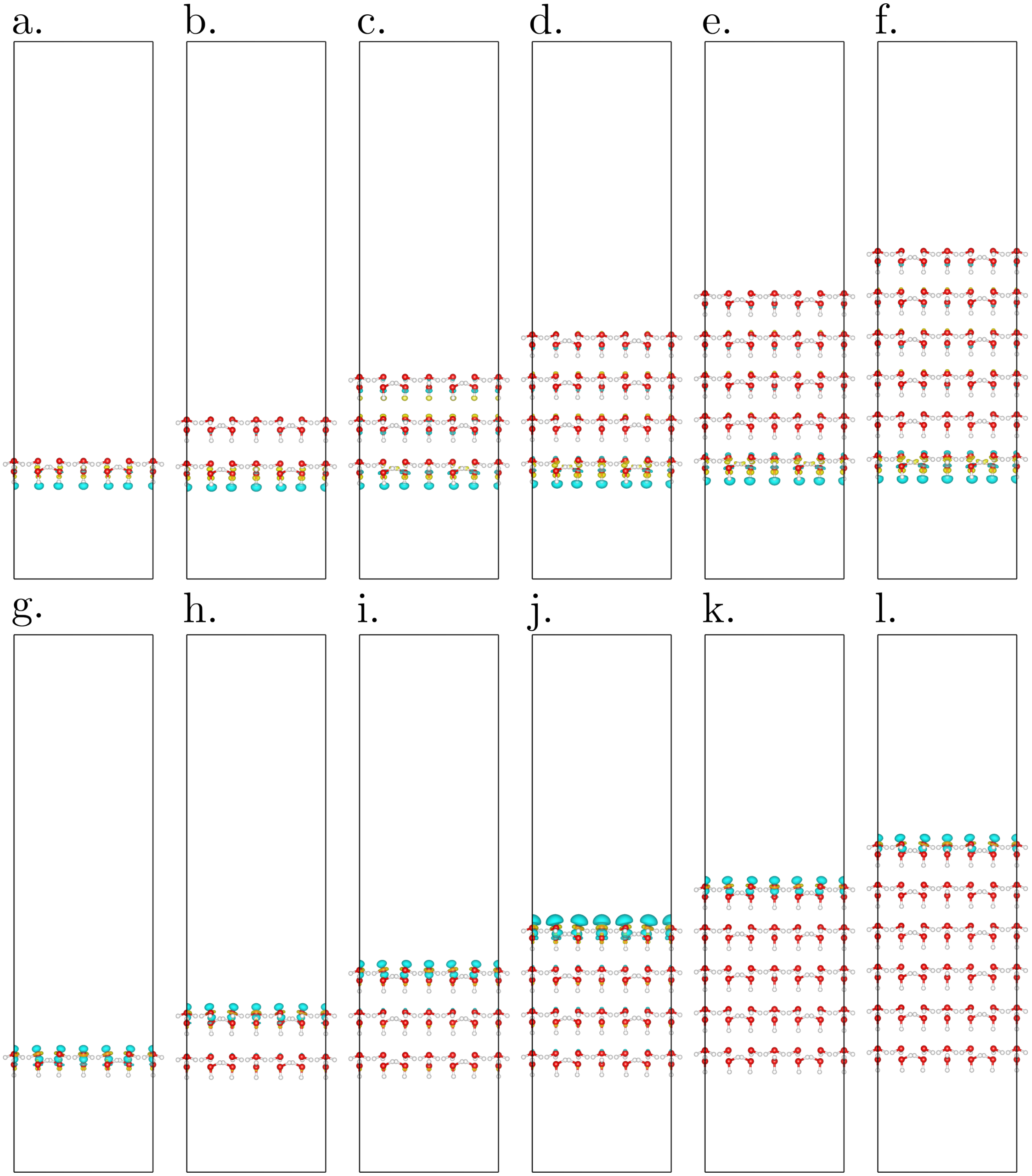

Figure S7: Fukui functions for the relaxed free-standing ice slabs. The positive and negative Fukui functions $f^{+}(\mathbf{r})$ and $f^{-}(\mathbf{r})$ are represented in (a-f) and (g-l), respectively. The equidensity values used for the representations are 2×10^{-6} and 4×10^{-6} for $f^{+}(\mathbf{r})$ and $f^{-}(\mathbf{r})$, respectively.

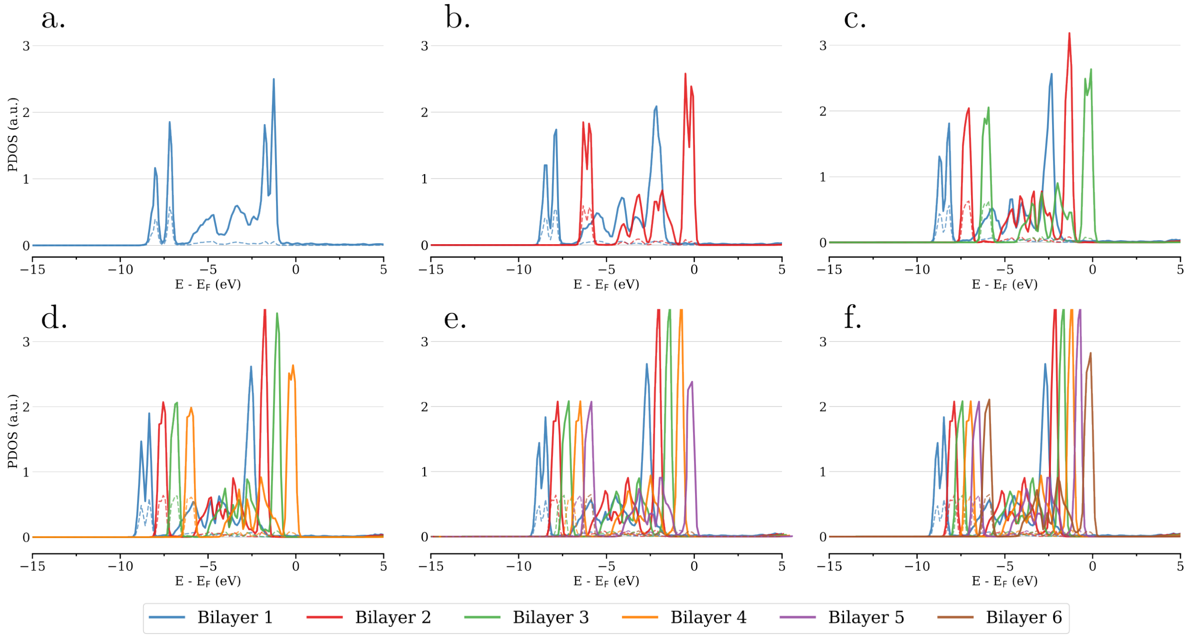

Figure S8: PDOS averaged per atom type and per bilayer for unrelaxed Au(111)-supported ice slabs in H-down configuration. Panels (a–f) correspond to systems comprising 1, 2, 3, 4, 5 and 6 bilayers, respectively. Solid lines represent oxygen atoms, and dashed lines represent hydrogen atoms.

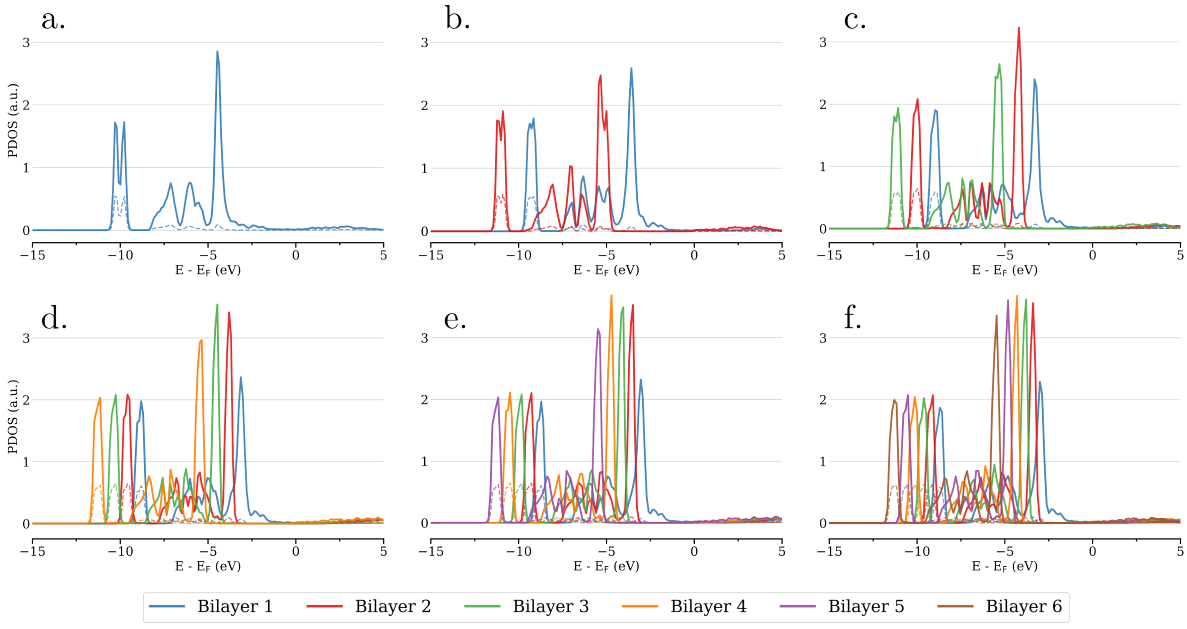

Figure S9: PDOS averaged per atom type and per bilayer for unrelaxed Au(111)-supported ice slabs in H-up configuration. Panels (a–f) correspond to systems comprising 1, 2, 3, 4, 5 and 6 bilayers, respectively. Solid lines represent oxygen atoms, and dashed lines represent hydrogen atoms.

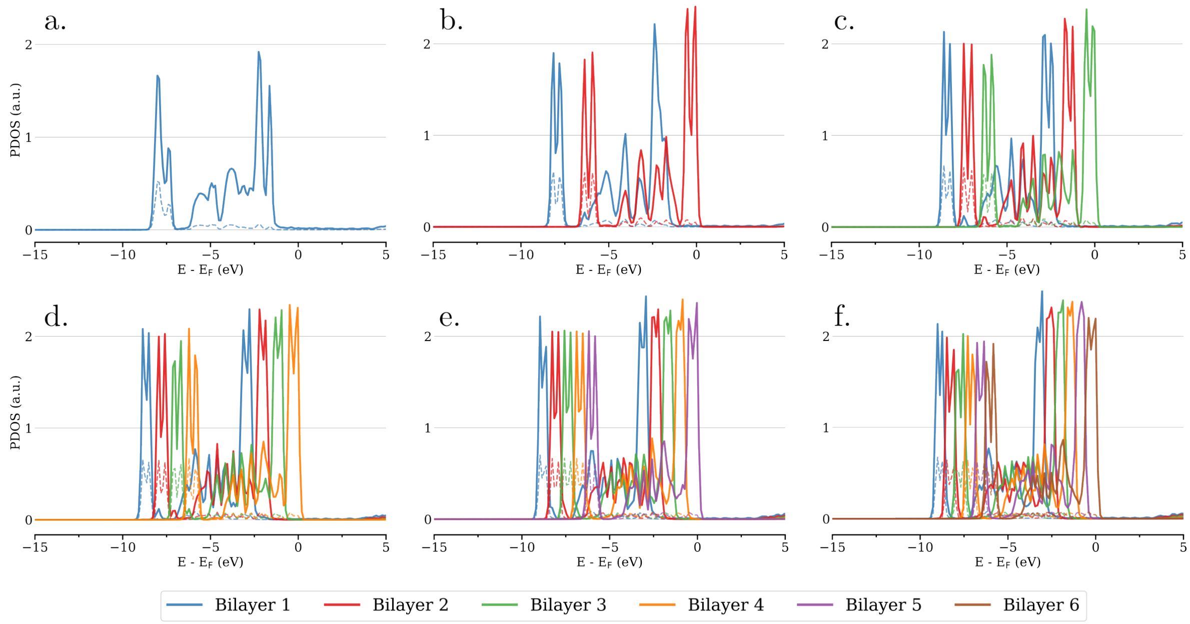

Figure S10: PDOS averaged per atom type and per bilayer for relaxed Au(111)-supported ice slabs in H-down configuration. Panels (a–f) correspond to systems comprising 1, 2, 3, 4, 5 and 6 bilayers, respectively. Solid lines represent oxygen atoms, and dashed lines represent hydrogen atoms.

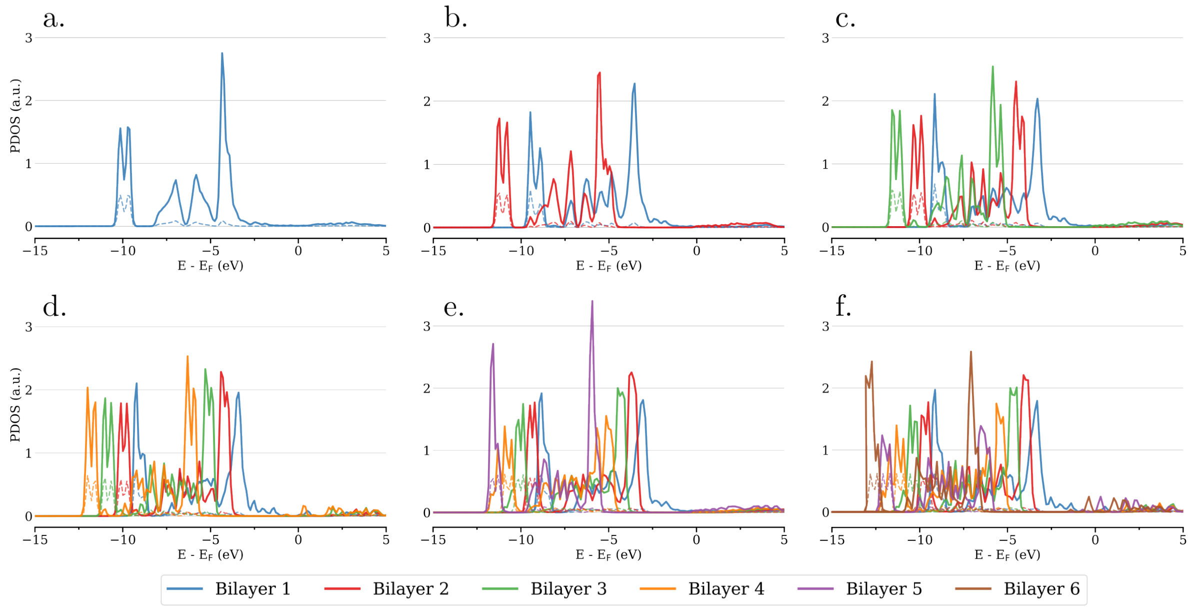

Figure S11: PDOS averaged per atom type and per bilayer for relaxed Au(111)-supported ice slabs in H-up configuration. Panels (a–f) correspond to systems comprising 1, 2, 3, 4, 5 and 6 bilayers, respectively. Solid lines represent oxygen atoms, and dashed lines represent hydrogen atoms.

Average out-of-plane dipole moment per molecule

For a dipole layer, the potential drop $\Delta\Phi_z$ across the layer (along the out-of-plane z-axis) is given by

$$\Delta\Phi_z = \frac{\mu_z}{\varepsilon_0} = \frac{p_z}{A\,\varepsilon_0} \tag{1}$$

where ε_0 is the vacuum permittivity, and μ_z is the layer's out-of-plane dipole moment per unit area. In the second equality, the latter was expressed in terms of the average out-of-plane dipole p_z and in-plane area A *per molecule*.

Within a water bilayer, each H_2O molecule occupies an area

$$A = 9\,\text{Å}^2 = 9 \times 10^{-20}\,\text{m}^2\ , \tag{2}$$

as determined from the simulation cells used in this study (see Figure S1), where 24 water molecules occupy a total in-plane area of 216 Å^2. Considering the conversion factor $1\,\text{D} = 3.33564 \times 10^{-30}$ C m and a vacuum permittivity $\varepsilon_0 = 8.854 \times 10^{-12}$ F/m, we obtain

$$\Delta\Phi_z = \frac{p_z\,(\text{D}) \times 3.33564 \times 10^{-30}}{(9 \times 10^{-20})(8.854 \times 10^{-12})} \approx (4.187\,\text{V})\ p_z\,(\text{D})\ . \tag{3}$$

According to our results, a single water bilayer induces a potential step of ~ 4.2 V (*cf.* main text, Figure 8a), corresponding to an average out-of-plane dipole p_z of approximately 1 D per water molecule.

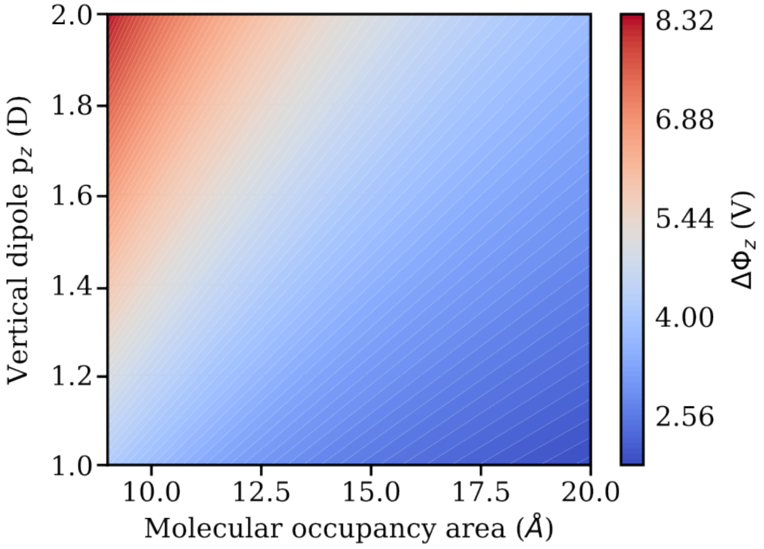

Figure S12: Evolution of the potential step $\Delta\Phi_z$ from Eq. (3) as a function of the out-of-plane dipole moment p_z and the molecular occupancy area A.

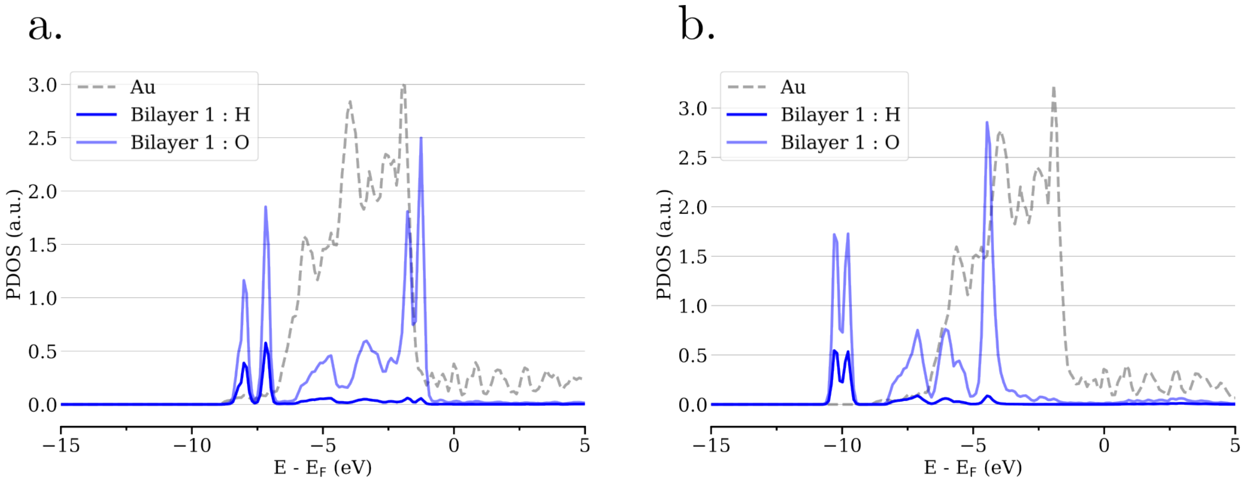

Figure S13: PDOS averaged per atom type for the Au(111)-supported single bilayer ice slab whereby (a) and (b) correpond to H-down and H-up configuration, respectively.
